# The relationship between physical activity and subjective wellbeing in infertile women: the mediating role of positive feedback and self-efficacy

**DOI:** 10.3389/fpubh.2026.1723969

**Published:** 2026-03-20

**Authors:** Mengxuan Liu

**Affiliations:** Jiangxi University of Technology, Nanchang, China

**Keywords:** infertile women, mediating role, physical activity, positive feedback, self-efficacy, subjective well-being

## Abstract

**Objective:**

This study examined the association between physical activity (PA) and subjective well-being (SWB) among infertile women and further investigated whether positive feedback (PF) and self-efficacy (SE) function as sequential mediators in this relationship.

**Methods:**

Data were collected from 954 infertile women recruited across several provinces in China using a cross-sectional design. PA, SWB, PF, and SE were measured through self-report questionnaires. A sequential mediation model was tested with PROCESS Model 6, and indirect effects were estimated using bootstrap procedures based on 5,000 resamples.

**Results:**

PA was positively associated with SWB in regression analyses (*β* = 0.101, *p* < 0.001). Both PF (indirect effect = 0.066) and SE (indirect effect = 0.066) independently mediated this association. A sequential pathway linking PA to SWB through PF and SE (PA → PF → SE → SWB) was also supported (indirect effect = 0.055). The total indirect effect accounted for 65% of the overall association.

**Conclusion:**

Higher levels of PA were associated with greater SWB among infertile women, and this association was partly explained by PF and SE operating in sequence. These findings indicate that incorporating structured physical activity alongside supportive feedback strategies may help enhance self-efficacy and improve psychological well-being.

## 1 Introduction

Infertility is widely recognized as a significant public health and social concern across the globe (1–3). Reports from the World Health Organization (WHO) (4, 5) indicate that global infertility prevalence ranges from approximately 8 to 12% (6, 7). In China, this issue is particularly severe, with infertility rates rising year by year; a study in Shanghai found the rate exceeding 15% (8). Unlike Western countries, China is deeply influenced by Confucian culture, which has long emphasized the traditional concepts of “passing on the family line” and “family continuity” (9). This makes infertile women not only face challenges in medical diagnosis and treatment but also endure dual pressures from family and society (10). This may lead them to be more prone to negative emotions such as anxiety and depression, and even suicidal behaviors (11–13). Subjective well-being represents an important indicator of overall life satisfaction and provides a multidimensional perspective for evaluating psychological health among infertile women (14). Existing research indicates that infertility can act as a proximal factor, leading to a loss of meaning in life and emotional emptiness among women, thereby significantly reducing their subjective wellbeing levels (15). Accordingly, the present study targets Chinese infertile women and examines their subjective well-being, given its theoretical relevance and practical implications. On one hand, it can address the urgent demands of Chinese social realities, providing scientific evidence to improve the psychological health and overall wellbeing of this group; on the other hand, it may provide additional evidence within a Chinese cultural context for research on reproductive health and psychological interventions.

### 1.1 Physical activity and subjective wellbeing

Physical activity (PA) encompasses various forms of exercise, including fitness training, recreational activities, health maintenance, and rehabilitation (16, 17), and psychological/cognitive training as their content, with the goals of enhancing physical fitness and mental health, as well as maintaining and improving bodily capabilities (18, 19); it has received considerable attention in global public health and mental health fields. Numerous studies indicate that regular PA can improve bodily functions, alleviate anxiety and depression, enhance self-esteem and self-regulation, and thereby promote psychological health and subjective wellbeing (20–23). From the perspective of Self-Determination Theory (SDT), autonomy, competence, and relatedness are considered fundamental psychological needs that are often frustrated in infertile women (24): fertility failures undermine competence and autonomy, while social pressures impair relatedness needs, leading to declines in life satisfaction and wellbeing. PA holds promise by fulfilling competence needs through exercise achievements (25), strengthening relatedness needs via group interactions, and reducing negative emotions through attention diversion and emotional regulation, thereby enhancing their SWB (26). Although there is substantial evidence in general populations and chronic disease patients, direct research on infertile women remains limited; existing findings suggest that PA may help improve their psychological states and wellbeing, but its specific pathways and mechanisms still require further elucidation. Based on this, this study proposes a hypothesis.

*H1*: PA is positively associated with infertile women’s SWB.

### 1.2 The mediating role of positive feedback

Understanding how PA relates to SWB in infertile women requires attention to psychosocial mechanisms that may connect these variables. One potential pathway is positive feedback (PF). In this study, PF refers to perceived recognition, affirmation, or encouragement from significant others regarding efforts to maintain health or cope with fertility-related stress. Although PA is often discussed in relation to social interaction and support (27, 28), participation in PA does not necessarily depend on group-based settings. Exercise may be performed individually while still being associated with perceived affirmation. Women who engage in PA may receive encouragement from partners, family members, healthcare professionals, or peers, and some may share health-related behaviors in online environments (29). In addition, maintaining an active lifestyle may strengthen perceptions of being responsible or valued, which may be reflected in PF (30). Previous studies have reported that higher levels of PF are linked to better emotional adjustment, greater resilience, and enhanced life satisfaction (31). Some studies also report positive associations between regular PA and perceived support or affirmation (32). PF may therefore represent a psychological mechanism linking PA and SWB in infertile women. Moreover, PF can foster more optimistic cognitive appraisals and strengthen adaptive psychological resources (33), thereby promoting SWB (34). Individuals who report higher levels of PF often experience greater life satisfaction, stronger self-regulation, higher self-worth (35), and fewer depressive symptoms (36). In the context of PA and psychological adaptation, prior research has suggested that PA may be associated with increased perceptions of high-quality positive feedback (37), which in turn predicts stronger resilience and wellbeing. Overall, existing evidence indicates that PF may function as a mediating pathway between PA and SWB.

*H2*: PF acts as a mediator in the relationship between PA and infertile women’s SWB.

### 1.3 The mediating role of self-efficacy

Self-efficacy (SE) refers to an individual’s belief in their ability to successfully cope with specific challenges to produce expected outcomes, which can help individuals maintain confidence and motivation when facing adversity (38). There are two main understandings of SE: the first views it as a stable personality trait, while the second tends to regard it as a dynamic capability that can be developed through experience and training; currently, the second understanding dominates empirical research. The enhancement of SWB in infertile women is closely linked to higher levels of SE (39). Infertile women need to adapt to fertility-related pressures or adversities in their social environments, which can help them mobilize resources through learning and practice to strengthen SE. The recreational, open, and competitive characteristics of PA contexts provide excellent opportunities for the development of SE in infertile women. In related research on SE development, PA is considered an important influencing factor. Regular PA can enhance SE through pathways that include boosting confidence in physical and psychological abilities, improving individuals’ physiological and psychological states, and acting as a “catalyst” for a sense of achievement (40). Individuals with higher levels of SE often experience positive psychological benefits. SE is closely connected to SWB; research has found that higher SE has been associated with greater life satisfaction and positive affect, whereas lower negative affect has also been reported among individuals with stronger SE. With structural equation models showing a significant direct effect of SE on SWB. Additionally, SE has been discussed as a mediating variable in numerous studies, such as its mediating role in the relationships between hope and mental health, as well as SWB (41).

*H3*: SE acts as a mediator in the relationship between PA and infertile women’s SWB.

### 1.4 Development of the sequential mediation framework

In SE theory, PF is considered an important external resource that supports individual SE (42). When PF is viewed as a resource, infertile women focus their attention on positive support to cope with challenges (43); thus, individuals with high levels of PF can gain a sense of control and efficacy when facing fertility difficulties (44). Research from the perspective of conservation of resources theory indicates that PF is closely related to SE; the higher the level of PF, the higher the individual’s SE (45, 46). Holden et al. (47) found that the level of basic psychological needs in SE theory is significantly positively correlated with individuals’ PF levels. The prerequisite for satisfying individuals’ basic psychological needs is that individuals must have a keen perception of their current environment and self-capabilities, enabling them to perform self-regulation more accurately, engage more strongly in chosen activities or behaviors, and thereby generate positive experiences. Individuals’ perceptions of their current environment and self-capabilities reflect the concept of PF (48). Empirical research also indicates that PF is significantly related to individuals’ efficacy behaviors; PF can promote self-reinforcement of behaviors, thereby facilitating SE or autonomous behaviors (49, 50). On the basis of these theoretical and empirical considerations, the following hypothesis was formulated:

*H4*: PF and SE are hypothesized to sequentially mediate the association between PA and SWB in infertile women.

**Figure 1:**
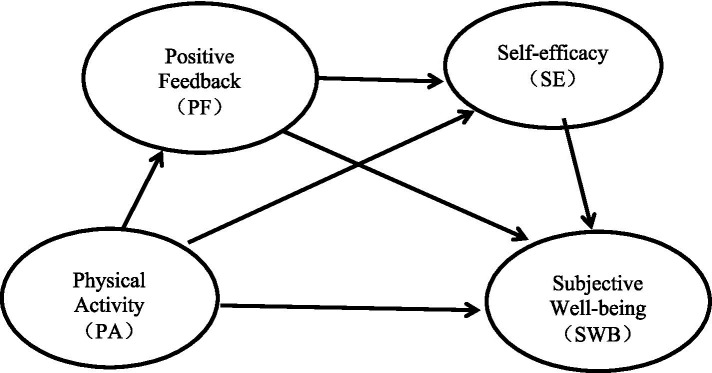
The hypothesized model diagram.

In summary, the present study investigates the association between PA and SWB among infertile women. Additionally, the study examines whether PF and SE mediate the association between PA and SWB. As illustrated in Figure 1, the study proposes a hypothetical model to uncover the underlying mechanisms linking PA with infertile women’s SWB. By exploring this model, this study not only offers a theoretical framework for understanding the association of PA with wellbeing in infertile populations but also provides a foundation for developing targeted interventions that promote PA alongside its related PF and SE to enhance long-term psychological health.

It should be noted that the directional specification of the proposed model is theory-driven. Based on self-determination theory, social support theory, and prior empirical research, PA was conceptualized as an antecedent behavioral factor associated with psychosocial resources and SWB. However, given the cross-sectional design of the present study, temporal ordering cannot be empirically established.

## 2 Materials and methods

### 2.1 Participants

This study employed a mixed recruitment strategy, combining targeted purposive sampling and convenience sampling, to identify and enroll women experiencing infertility. To enhance sample diversity and representativeness, recruitment was conducted in five provinces in mainland China, namely Jiangxi, Hunan, Guangdong, Zhejiang, and Fujian.

Recruitment was divided into two distinct phases to ensure methodological rigor. First, a pilot survey was conducted from January to February 2025 to test feasibility, refine the questionnaire, and evaluate recruitment strategies. During this phase, we collaborated with reproductive medicine departments in two hospitals per province and utilized online platforms, including WeChat-based infertility support mutual-aid groups and forums. Community networks, such as women’s health workshops and support groups organized by local non-governmental organizations, were also leveraged. Potential participants were approached through informational posters, email invitations, and social media announcements, with eligibility screened via a brief online pre-qualifier form. The pilot phase collected preliminary data from 30 participants, among whom 12 were invited for interviews to assess content appropriateness. These data guided adjustments to questionnaire wording, adaptability, and cultural sensitivity.

The formal data collection phase ran from March to September 2025, expanding recruitment based on pilot insights. We extended collaborations to include additional hospitals (totaling 15 across provinces) and digital channels, such as Douyin (TikTok) and Xiaohongshu infertility awareness communities. Research assistants, trained in advance, conducted in-person outreach at clinics (with permission from department heads), while online recruitment used targeted ads and snowball sampling. No incentives were provided to avoid coercion, emphasizing voluntary participation.

In the formal survey, a total of 996 questionnaires were distributed. After excluding invalid responses (e.g., incomplete submissions or those failing attention checks), 954 valid responses were retained, yielding an effective response rate of 95.78%. A priori power analysis (using G*Power 3.1; α = 0.05, power = 0.80, effect size f^2^ = 0.15, based on prior mediation studies) indicated a minimum sample size of 742 for detecting medium-sized effects in multiple regression analyses, which our final sample size exceeded.

Participants were required to meet all eligibility criteria: (1) female, aged 18 years or older; (2) clinically diagnosed with infertility (defined per WHO guidelines as failure to conceive after ≥12 months of regular unprotected intercourse); (3) permanent resident in one of the target provinces; and (4) able to read and independently complete the digital questionnaire in Mandarin. Exclusion criteria included self-reported severe physical illnesses (e.g., cancer requiring ongoing treatment), cognitive impairments (e.g., dementia), or psychiatric histories (e.g., schizophrenia), which could confound responses or compromise informed consent.

All data were collected via an anonymous online survey platform (Wenjuanxing, a secure Chinese equivalent to Qualtrics) to minimize social desirability bias and protect privacy. The questionnaire took approximately 15–20 min to complete and included validated scales for physical activity, positive feedback, self-efficacy, and subjective wellbeing, with demographic items at the end.

Prior to accessing the questionnaire, participants viewed an electronic informed consent form detailing the study’s purpose, voluntary nature, data confidentiality, potential minimal risks (e.g., emotional discomfort from fertility-related questions), and the right to withdraw at any time. Only those providing electronic consent via a checkbox could proceed. To ensure data quality, we implemented rigorous checks: (1) completeness verification (excluding surveys with >20% missing data); (2) response consistency (e.g., reverse-scored items and attention checks, such as “Please select ‘agree’ here”); (3) outlier detection (e.g., implausibly short completion times <5 min); and (4) duplicate IP screening. Invalid cases were removed blindly by two independent coders, with inter-rater agreement >95%.

The study protocol was approved by the Institutional Review Board (IRB) of the authors’ affiliated university (IRB-JXUT-PEC-20241208). All procedures adhered to the Declaration of Helsinki and Chinese ethical guidelines for human subjects research. Participant anonymity was maintained throughout, with data stored on password-protected servers accessible only to the research team. De-identified datasets will be made available upon reasonable request post-publication to promote transparency.

### 2.2 Measures

#### 2.2.1 Physical activity

PA was measured with the Chinese version of the Physical Activity Rating Scale revised by Liang (51). The scale includes three items assessing exercise intensity, frequency, and duration. Each item is rated on a five-point scale ranging from 1 to 5, with higher scores indicating higher levels of activity. For instance, the intensity item asks about the usual level of physical activity, scored from 1, representing no or light activity, to 5, representing very vigorous exercise. The PARS-3 has been widely applied in Chinese samples (12, 52, 53). The Cronbach’s alpha coefficient in this sample was 0.717. The scale evaluates intensity, frequency, and duration of exercise, but it does not differentiate between group-based and individual forms of physical activity.

#### 2.2.2 Subjective well-being

SWB was measured with the Chinese adaptation of the Campbell Index of Well-Being developed by Campbell and colleagues (54). The scale has been widely applied in research on psychological and social functioning. In this survey, minor wording modifications were made during the pilot phase to improve cultural suitability for infertile women experiencing social pressure. The instrument contains nine items representing two components, including an eight-item general affect index and a single-item life satisfaction index. Affect items are rated on a seven-point semantic differential scale, while the life satisfaction item uses a seven-point Likert-type format. The total score is calculated by weighting the general affect index by 1.0 and the life satisfaction index by 1.1, producing a possible range of approximately 2.1 to 14.7, with higher scores indicating better subjective well-being. The Cronbach’s alpha coefficient in this sample was 0.864.

#### 2.2.3 Positive feedback

Perceived PF was measured with the Positive Feedback Scale developed by Liu et al. (55). The scale includes six items reflecting individuals’ perceptions of recognition and encouragement from people around them. Responses are provided on a five-point Likert scale ranging from 1, strongly disagree, to 5, strongly agree. A sample item is “I feel that people around me recognize my efforts to improve my health.” Item scores are summed to obtain an overall index, with higher scores representing stronger perceived positive feedback. The Cronbach’s alpha coefficient in this sample was 0.776.

#### 2.2.4 Self-efficacy

SE was measured using the General Self-Efficacy Scale developed by Schwarzer et al. (56). The instrument consists of ten items assessing individuals’ confidence in handling challenging situations. Each item is rated on a four-point response scale ranging from 1, not at all true, to 4, exactly true. A representative item is “I can always manage to solve difficult problems if I try hard enough.” Mean scores were computed within a possible range of 1 to 4, and higher scores reflect stronger self-efficacy. The Cronbach’s alpha coefficient in this sample was 0.873.

### 2.3 Statistical analysis

All statistical analyses were conducted using IBM SPSS version 26.0. For continuous variables, normally distributed data were summarized by their means and standard deviations (M ± SD), whereas non-normally distributed variables were expressed using medians and interquartile ranges [M (P25, P75)]. To assess potential common method bias (CMB), Harman’s single-factor test was conducted by entering all measurement items into an unrotated exploratory factor analysis. Subsequently, Pearson’s correlation coefficients were computed to examine bivariate associations among PA, PF, SE, and SWB. To further clarify the underlying mechanisms, a serial mediation analysis was carried out using the PROCESS macro (Model 6) developed by Hayes, estimating both direct and indirect effects within the regression framework. The mediating functions of PF and SE were verified with bootstrap resampling procedures (5,000 iterations) and 95% bias-corrected confidence intervals (CIs). An indirect effect was considered statistically significant if zero did not fall within the confidence interval.

## 3 Results

### 3.1 Preliminary analyses

To examine potential common method variance, all measurement items were entered into an unrotated exploratory factor analysis. Six factors with eigenvalues greater than 1 emerged. The first factor accounted for 33.873% of the total variance, which is below the commonly referenced 40% criterion, suggesting that common method bias is unlikely to pose a serious concern in the present dataset. Although Harman’s single-factor approach has been criticized for its limited sensitivity, it remains a widely used preliminary diagnostic procedure in survey-based research. More advanced techniques, such as unmeasured latent method factor (ULMF) modeling or marker-variable approaches within a structural equation modeling framework, were not applied because the mediation analysis was conducted using regression-based PROCESS procedures rather than latent variable modeling. Future studies employing confirmatory factor analysis may adopt these more rigorous strategies to further assess potential method effects.

Prior to conducting correlation and mediation analyses, all variables demonstrated approximately normal distributions (skewness ranged from 0.06 to 1.61; kurtosis ranged from −1.12 to 1.64). Pearson correlation analyses were subsequently performed to examine associations among PA, PF, SE, and SWB. Inter-construct correlations ranged from 0.486 to 0.768. Descriptive statistics and correlation coefficients are presented in Table 1. Although the correlation between SE and SWB was relatively high (r = 0.768), it remained below conservative thresholds (e.g., 0.85) commonly used to indicate serious discriminant validity concerns. The constructs are theoretically distinct, with self-efficacy reflecting perceived capability beliefs and subjective wellbeing reflecting evaluative and affective life assessments. Future research employing confirmatory factor analysis and formal HTMT procedures would allow for a more rigorous assessment of discriminant validity.

**Table 1:** Descriptive statistics and correlation matrix.

Variable	*M*	*SD*	1	2	3	4
1. PA	16.69	22.91				
2. PF	2.75	0.98	0.512^***^			
3. SE	2.44	0.73	0.486^***^	0.742^***^		
4. SWB	7.36	3.19	0.531^***^	0.701^***^	0.768^***^	

***p < 0.001.

### 3.2 Demographic characteristics

The final valid sample comprised 954 infertile women from five provinces in China (Jiangxi, Hunan, Guangdong, Zhejiang, and Fujian), all clinically diagnosed with infertility. Participants’ ages ranged from 24 to 42 years (M ± SD = 29.73 ± 6.11), with 138 (14.5%) under 30 years, 426 (44.7%) aged 31–35 years, and 390 (40.8%) aged 36 years or older. The sample was diverse in marital status, education, occupation, income, and residence, reflecting a broad representation of infertile women in urban and rural settings. Categorical variables were summarized using frequencies and percentages, while continuous variables (e.g., age) were reported as means and standard deviations. Detailed demographic information is presented in Table 2.

**Table 2:** Demographic characteristics of the sample (*N* = 954).

Characteristics	Categories	*N (%)*	*M ± SD*
Marital status	Married	551 (57.8)	
	Single	235 (24.6)	
	Divorced	130 (13.6)	
	Other	38 (4)	
Educational level	Elementary school or below	17 (1.8)	
	Middle school	374 (39.2)	
	High school/Vocational school	374 (39.2)	
	Associate/Bachelor’s degree	153 (16)	
	Master’s degree or above	36 (3.8)	
Occupational status	Full-time employment	133 (13.9)	
	Part-time employment	315 (33)	
	Homemaker	258 (27)	
	Unemployed/Not currently working	248 (26)	
Monthly income	<3,000 RMB	241 (25.3)	
	3,000–6,000 RMB	239 (25.1)	
	6,001–10,000 RMB	225 (23.6)	
	>10,000 RMB	249 (26.1)	
Place of residence	Urban	568 (59.5)	
	Rural	386 (40.5)	
Age	954		29.73 ± 6.11

### 3.3 Chain mediation effect analysis

Prior to conducting mediation analysis, multicollinearity diagnostics indicated acceptable levels (VIF values ranged from 1.176 to 1.490). After controlling for marital status, education level, occupational status, monthly income, and residence, regression analysis showed that physical activity significantly positively predicted positive feedback (b = 0.013, *β* = 0.294, SE = 0.001, t = 9.732, *p* < 0.001), and the model’s explained variance was R^2^ = 0.175 (*F* = 33.381, *p* < 0.001). Physical activity significantly positively predicted self-efficacy (b = 0.005, *β* = 0.171, SE = 0.001, t = 6.008, *p* < 0.001), and positive feedback significantly positively predicted self-efficacy (b = 0.362, *β* = 0.491, SE = 0.022, t = 16.783, *p* < 0.001), with the model explaining 33.1% of the variance (R^2^ = 0.331, *F* = 66.783, *p* < 0.001). Additionally, physical activity significantly positively predicted subjective wellbeing (b = 0.014, *β* = 0.101, SE = 0.004, t = 3.751, *p* < 0.001), positive feedback significantly positively predicted subjective wellbeing (b = 0.728, *β* = 0.224, SE = 0.100, t = 7.280, *p* < 0.001), and self-efficacy significantly positively predicted subjective wellbeing (b = 1.692, *β* = 0.384, SE = 0.132, t = 12.775, *p* < 0.001). The overall model accounted for 42.9% of the variance in subjective wellbeing (R^2^ = 0.429, *F* = 88.617, *p* < 0.001). See Table 3 for details.

**Table 3:** Regression analysis results.

Dependent variable	Independent variable	*b*	*β*	*SE*	*t*	*R^2^*	*F*
PF	Marital status	0.147	0.150	0.025	5.921^***^	0.175	33.381^***^
	Educational level	−0.015	−0.015	0.035	−0.431		
	Occupation	−0.097	−0.098	0.030	−3.327^**^		
	Monthly income	−0.014	−0.014	0.026	−0.538		
	Place of residence	0.156	0.159	0.061	2.584^*^		
	PA	0.013	0.294	0.030	9.732^***^		
SE	Marital status	0.001	0.002	0.023	0.087	0.331	66.783^***^
	Educational level	0.041	0.057	0.031	1.838		
	Occupation	0.007	0.009	0.027	0.350		
	Monthly income	−0.020	−0.028	0.024	−1.164		
	Place of residence	0.012	0.016	0.056	0.294		
	PA	0.005	0.171	0.029	6.008^***^		
	PF	0.362	0.491	0.029	16.783^***^		
SWB	Marital status	0.380	0.119	0.021	5.555^***^	0.429	88.617^***^
	Educational level	0.070	0.022	0.029	0.765		
	Occupation	−0.167	−0.052	0.025	−2.113^*^		
	Monthly income	−0.064	−0.020	0.022	−0.911		
	Place of residence	0.289	0.090	0.051	1.758		
	PA	0.014	0.101	0.027	3.751^***^		
	PF	0.728	0.224	0.031	7.280^***^		
	SE	1.692	0.384	0.030	12.775^***^		

*p < 0.05, **p < 0.01, ***p < 0.001.

Mediation tests using 5,000 Bootstrap resamples revealed that the mediation effects consisted of three indirect paths: (1) the individual mediation of PF (indirect effect 1); (2) the individual mediation of SE (indirect effect 2); (3) the chain mediation of “PF to SE” (indirect effect 3). The 95% confidence interval for indirect effect 1 was [0.044, 0.092], for indirect effect 2 was [0.041, 0.092], and for indirect effect 3 was [0.040, 0.075], none of which contained zero, indicating all three indirect effects were significant. See Table 4.

**Table 4:** Mediation effects and proportions.

Path	Effect	Boot SE	Boot LLCI	Boot ULCI	Effect proportion
Total effect	0.288	0.03	0.229	0.347	100%
Direct effect	0.101	0.027	0.048	0.154	35%
Total indirect effect	0.187	0.019	0.153	0.227	65%
Ind1	0.066	0.012	0.044	0.092	23%
Ind2	0.066	0.013	0.041	0.092	23%
Ind3	0.055	0.009	0.04	0.075	19%

The table outlines the direct and indirect impacts of PA, PF, and SE on infertile women’s SWB. Total effect represents the direct and indirect impacts of PA on SWB. The indirect effects, respectively, were calculated via the bias-corrected percentile bootstrap approach. Ind1: PA → PF → SWB; Ind2: PA → SE → SWB; Ind3: PA → PF → SE → SWB.

**Figure 2:**
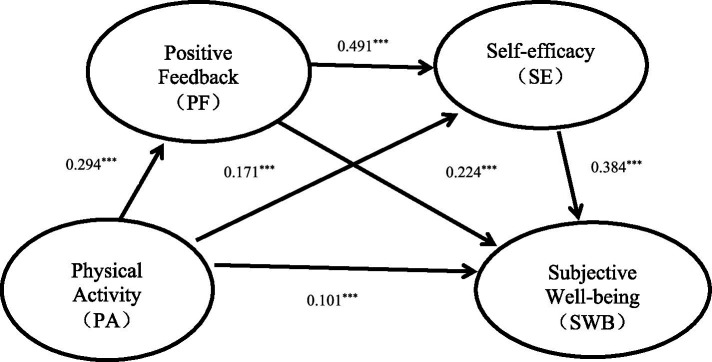
Mediation effect diagram. ***p < 0.001.

The overall mediation analysis results (see Figure 2) showed that PF and SE played significant mediating roles between PA and SWB, with a total indirect effect of 0.187, accounting for 65% of the total effect. Among them: the individual mediation effect of PF was 0.066, accounting for 23%; the individual mediation effect of SE was 0.066, accounting for 23%; the chain mediation effect of “PF and SE” was 0.055, accounting for 19%. The 95% confidence intervals for the above indirect effects did not include zero, indicating statistical significance.

## 4 Discussion

The present research developed and tested a sequential mediation framework to clarify how physical activity relates to subjective well-being among infertile women. The findings indicate that the association between PA and SWB operates not only directly but also indirectly through perceived positive feedback and self-efficacy, with evidence supporting a sequential pathway linking these psychosocial factors. Theoretically, this helps clarify potential mechanisms linking PA and SWB in infertile women, enriching the theoretical research outcomes in this field; practically, it facilitates multi-faceted approaches to proactively design intervention strategies, such as psychological support programs incorporating PA, providing empirical support for enhancing SWB levels in infertile women.

### 4.1 The relationship between physical activity and subjective wellbeing among infertile women

The results demonstrated a positive association between PA and SWB in infertile women, which is consistent with prior empirical evidence (33, 34, 57). From a positive psychology perspective, engagement in PA may foster psychological resilience, which may help buffer the negative impact of fertility-related stress on mental health (58, 59). Furthermore, regular PA may be related to improved neuroendocrine balance, enhanced emotional regulation abilities, and expanded support networks (60, 61). A large body of evidence supports a stable positive association between PA and SWB, particularly pronounced in vulnerable groups such as the older adults and individuals with disabilities (62); this study provides evidence for the association between PA and SWB in the group of infertile women, further extending its application in reproductive health contexts, indicating that PA may be relevant in alleviating fertility-related self-uncertainty and emotional burdens (such as anxiety and depression) (63, 64).

### 4.2 The independent mediating role of positive feedback

The analysis further revealed that PF functioned as an intermediary variable linking PA and SWB. In the present research, PF refers to perceived recognition or encouragement from significant others regarding one’s efforts to maintain health or cope with fertility-related stress. Importantly, PF represents individuals’ perceived social affirmation, which may arise beyond the immediate exercise context. Although PA is often discussed in connection with social interaction (65–68), the current study did not assess whether exercise was performed in group-based or solitary contexts. The PARS-3 instrument measures exercise intensity, frequency, and duration but does not distinguish between group-based and individual activity formats. Therefore, the specific social setting in which PF was experienced cannot be determined. Participation in PA does not necessarily require direct interpersonal interaction (69–71). Even when exercise is undertaken individually, women may still perceive affirmation from partners, family members, healthcare providers, or online communities who recognize their health-related efforts. PF reflects a perception of support and positive evaluation, and prior research has linked higher PF to better emotional adjustment, stronger self-regulation, and greater life satisfaction (72–74). In this framework, PF may represent one perceived psychosocial pathway through which PA is associated with SWB. However, given the cross-sectional design and the absence of activity format measurement, causal conclusions cannot be drawn.

### 4.3 The independent mediating role of self-efficacy

Similarly, SE emerged as a significant mediator in the relationship between PA and SWB. This result is consistent with previous research findings, namely that PA was positively associated with SE and SE was positively associated with SWB (75). One possible explanation is that regular participation in PA fulfills individuals’ needs for competence and mastery; individuals with regular exercise habits have a tendency toward self-realization, and active participants typically exhibit higher confidence and motivation, leading to higher SE levels; additionally, through repeated experiences of achievement in exercise, individuals can strengthen their beliefs in their own abilities, reduce feelings of helplessness caused by fertility stress, and thereby maintain positive attitudes and emotional stability when facing challenges. In this context, PA may be associated with infertile women’s SWB through SE (76, 77).

### 4.4 The chain mediating role of positive feedback and self-efficacy

Moreover, the results supported a sequential mediation pathway in which PA was linked to enhanced PF, which in turn strengthened SE and ultimately contributed to higher SWB. Specifically, PA was related to SWB through higher perceived positive feedback and, subsequently, stronger self-efficacy. PF can be understood as a perceived social resource characterized by recognition and encouragement from significant others. Such affirmation may help infertile women feel supported in coping with fertility-related stress and may foster greater confidence and motivation. Previous research has shown that perceived support and positive appraisal are associated with better psychological adjustment and fulfillment of basic psychological needs (78, 79). Individuals who perceive higher levels of PF may also demonstrate better emotional regulation and greater self-reflection (80–82), which can contribute to stronger efficacy beliefs. Although the direct association between PA and SWB was modest (*β* = 0.101), a considerable portion of the overall association was statistically accounted for by indirect pathways (65%). It should be noted that this proportion reflects the decomposition of regression-based effects within a cross-sectional framework rather than the strength of a causal mechanism. The findings suggest that psychosocial variables may represent important statistical pathways linking PA and wellbeing; however, given the correlational design and the conceptual proximity among psychological constructs, the magnitude of the indirect effects should be interpreted with appropriate caution. This suggests that the statistical association between PA and wellbeing was largely accounted for by psychosocial variables in the present model. Engaging in regular PA may be linked to a more accepting and confident self-view, which is associated with higher perceived feedback and stronger self-efficacy, ultimately relating to improved subjective wellbeing (83).

### 4.5 Implications and limitations

This study, grounded in social support theory and self-determination theory, examined the association between PA and SWB in infertile women and explored its potential mechanisms (84). Most previous research on infertile women has focused on biological or reproductive outcomes, whereas psychological wellbeing has received comparatively less attention. The present findings contribute to this area by highlighting psychosocial pathways that may link health behaviors with wellbeing. The results also provide empirical support for the relevance of social support and self-determination perspectives in understanding psychological adjustment among infertile women. Practically, these findings underscore the potential value of incorporating PA into supportive interventions aimed at enhancing psychological adaptation in this population (85, 86).

This study has several limitations. First, PA was assessed using self-reported data from the PARS-3 scale. Although this instrument is widely used, self-report measures are susceptible to recall bias and social desirability effects. Future studies may incorporate objective assessments, such as accelerometers or wearable devices, to improve measurement precision.

Second, the study did not distinguish between group-based and solitary forms of physical activity, nor did it assess specific activity contexts. Consequently, the contextual sources through which positive feedback was experienced cannot be directly identified. Future research should differentiate activity formats and examine whether social-contextual characteristics of PA influence the PA–PF association.

Third, the cross-sectional design precludes firm conclusions regarding causality. The directional specification of the model was theory-driven rather than based on empirically verified temporal ordering. Reverse or bidirectional associations are plausible; for example, individuals with higher subjective wellbeing may be more inclined to engage in regular physical activity, and depressive symptoms have been associated with reduced PA participation. Longitudinal or experimental research is needed to clarify temporal sequencing and potential reciprocal effects.

Fourth, participants were recruited from five provinces in China, which may limit generalizability to other regions or cultural contexts. The absence of a comparison group (e.g., fertile women) also restricts conclusions regarding the specificity of the observed associations. Future studies should include more diverse samples and appropriate comparison groups.

Fifth, several infertility-related clinical characteristics, including duration of infertility and current treatment status, were not assessed. These factors may be associated with psychological adjustment and wellbeing, and their omission means that residual confounding cannot be completely excluded. Accordingly, the observed associations between physical activity and subjective wellbeing should be interpreted with appropriate caution. Future investigations should incorporate more comprehensive clinical indicators to determine whether the reported relationships remain stable after accounting for infertility-related heterogeneity.

Finally, although Harman’s single-factor test did not suggest substantial common method variance, this approach provides only a preliminary diagnostic assessment. More advanced procedures, such as unmeasured latent method factor (ULMF) modeling or marker-variable techniques within a structural equation modeling framework, were not implemented in the present study. Future research employing latent variable modeling may offer a more rigorous evaluation of potential method effects.

## 5 Conclusion

In summary, the current cross-sectional investigation involving 954 infertile women in China explored how PA relates to SWB, emphasizing the sequential mediating roles of PF and SE. Using validated scales and PROCESS Model 6 mediation analysis (with 5,000 Bootstrap resamples), PA was positively associated with SWB, PF and SE independently mediated this relationship, and a chain mediation pathway was supported, with the mediation accounting for 65%. Overall, the evidence indicates that PA contributes to SWB through both direct effects and indirect influences via PF and SE. Implications include integrating PA into clinical interventions, combined with feedback-oriented support to enhance SE and support psychological health in infertile women.

## Data Availability

The raw data supporting the conclusions of this article will be made available by the authors, without undue reservation.
